# The value of detecting immunoglobulin gene rearrangements in the diagnosis of B-cell lymphoma

**DOI:** 10.18632/oncotarget.20330

**Published:** 2017-08-18

**Authors:** Can Lu, QiuYan He, Wei Zhu, ChunYan Fu, JianHua Zhou, YongGuang Tao, Shuang Liu, DeSheng Xiao

**Affiliations:** ^1^ Department of Pathology, Xiangya Hospital/ School of Basic Medicine, Central South University, Changsha, Hunan 410078, China; ^2^ Cancer Research Institute, School of Basic Medicine, Central South University, Changsha, Hunan 410078, China; ^3^ Key Laboratory of Carcinogenesis and Cancer Invasion, Ministry of Education, Hunan 410078, China; ^4^ Key Laboratory of Carcinogenesis, National Health and Family Planning Commission, Hunan 410078, China; ^5^ Center for Medicine Research, Xiangya Hospital, Central South University, Changsha, Hunan 410078, China; ^6^ Department of Pathology, Xiangya Hospital, School of Basic Medicine, Central South University, Changsha, Hunan 410078, China

**Keywords:** immunoglobulin, gene rearrangement, B-cell lymphoma

## Abstract

**Objective:**

To discuss the clinical value of immunoglobulin gene rearrangements in the diagnosis of B-cell lymphoma.

**Methods:**

A total of 209 cases of B-cell lymphomas and 35 cases of reactive lymphoid hyperplasia were selected for DNA extraction and PCR amplification using the BIOMED-2 primer system. Gel electrophoresis of heteroduplexes was used to analyze immunoglobulin gene rearrangements.

**Results:**

A total of 209 cases of B-cell lymphoma, including 69 extranodal marginal zone B-cell lymphomas of mucosa-associated lymphoid tissue, 63 diffuse large B-cell lymphomas, 39 follicular lymphomas, 15 small lymphocytic lymphomas, 6 plasmacytomas, 6 mantle cell lymphomas, 7 nodal marginal zone B-cell lymphomas, and 4 lymphoplasmacytoid lymphomas, were examined. Immunoglobulin gene rearrangements were found in all 209 cases, with 93 IGHA, 122 IGHB, 98 IGHC, 167 IGK, 100 IGL, 167 IGHA/B/C, 204 IGH/IGK, 209 IGH/IGK/IGL, 129 IGH+IGK, 81 IGH+IGL, 83 IGK+IGL and 68 IGH+IGK+IGL gene rearrangements. Immunoglobulin gene rearrangements were not found in the 35 cases of reactive lymphoid hyperplasia. IGH and IGK gene rearrangements were mainly found in mantle cell lymphomas, small lymphocytic lymphomas, extranodal marginal zone B-cell lymphomas of mucosa-associated lymphoid tissue and diffuse large B-cell lymphomas. The IGH gene rearrangement was mainly found in lymphoplasmacytoid lymphomas and follicular lymphomas. IGK and IGL gene rearrangements were mainly found in plasmocytoma, and the IGK gene rearrangement was mainly found in nodal marginal zone B-cell lymphomas.

**Conclusions:**

The BIOMED-2 standardized immunoglobulin gene rearrangement detection system is an important tool in B-cell lymphoma diagnosis. Analysis of IGH, IGK and IGL gene rearrangements is valuable in confirming the classification of B-cell NHL.

## INTRODUCTION

Lymphoma is a malignant tumor derived from lymphoid tissue and lymph nodes, which can be classified into two types, Hodgkin lymphoma (HL) and non-Hodgkin lymphoma (NHL) [1, 2]. NHL is regarded as one of the most difficult diseases in clinical pathologic diagnosis due to its complexity and heterogeneity with a high misdiagnosis rate in differential diagnosis. In the late 1980s, gene analysis was utilized as an auxiliary diagnostic method together with histopathology and immunophenotyping analysis, as it could provide an alternative way for the diagnosis of malignant clones of lymphocytes. Immunohistochemistry and morphological analysis provided important value in distinguishing the immunological phenotypes of lymphomas, but they provided less information in the diagnosis of controversial cases. With the development of molecular biology, gene analysis has become an important supplementary method in the diagnosis of malignant lymphoma. Detection of gene rearrangements in immunoglobulin (IG), a specific marker of B lymphocyte clones, is an important method in the diagnosis of B-cell lymphoma [3–7]. In this study, to explore the clinical value of IG gene rearrangements in the diagnosis of B-cell lymphoma, the BIOMED-2 primer system was used for the analysis of IG gene rearrangements.

## RESULTS

### Classification of B-cell lymphoma

According to the expression status of different immune phenotypes, morphological analysis and classification standards of lymphatic and hematopoietic system tumors formulated by the WHO in 2015 [4], the selected 209 cases with B-cell lymphoma were classified again by two pathologists. The classification results showed that there were 69 extranodal marginal zone B-cell lymphomas of mucosa-associated lymphoid tissue (MALT lymphomas), 63 diffuse large B-cell lymphomas (DLBCL), 39 follicular lymphomas (FL), 15 small lymphocytic lymphomas (SLL), 6 plasmacytomas, 6 mantle cell lymphomas (MCL), 7 nodal marginal zone B-cell lymphomas, and 4 lymphoplasmacytoid lymphomas. These results are consistent with the previous diagnosis.

### Results of IG gene rearrangement analysis

IG gene rearrangements were found in all 209 B-cell lymphoma samples (Table 1), which included 93 IGHA, 122 IGHB, 98 IGHC, 135 IGH A/B, 167 IGH A/B/C (167/209, 80%), 129 IGKA, 109 IGKB, 167 IGKA/B (167/209, 80%), 100 IGL (100/209, 48%), 204 IGH/IGK (204/209, 98%), 186 IGH/IGL (186/209, 89%), 184 IGK/IGL (184/209, 88%), and 209 IGH/IGK/IGL (209/209, 100%) gene rearrangements. IG gene rearrangements were not found in the 35 samples of reactive lymphoid hyperplasia.

**Table 1:** IG gene rearrangements in all 209 B-cell lymphoma samples

Lymphoma type	n	IGHA	IGH A/ B	IGH A/B/ C	IGH/IGK	IGH/IGK/ IGL
MALT	69	32	45	57	67	69
DLBCL	63	28	44	51	61	63
FL	39	16	24	30	38	39
CLL	15	8	11	14	15	15
plasmocytoma	6	2	2	3	6	6
MCL	6	3	4	6	6	6
MZL	7	2	2	2	7	7
LPL	4	2	3	4	4	4
Total	209	93	135	167	204	209

### Correlation between IG gene rearrangements and lymphoma classification

**Figure 1:**
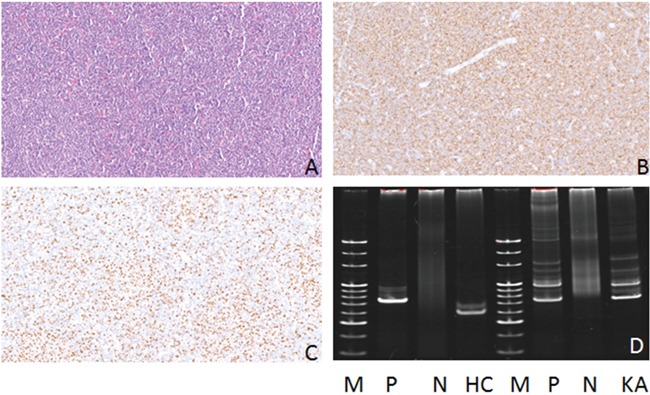
Hematoxylin-eosin (HE), Immunohistochemical staining and IG gene rearrangement of mantle cell lymphomas **(A)** Mantle cell lymphomas is composed of simplex small to medium sized lymphoid cells, with diffuse distribution pattern(HE) (×200); **(B)** The lymphoma cells were positive for CD20 protein in the cytomembrane (×200); **(C)** The lymphoma cells were positive for CyclinD1 protein in the nucleus (×200); **(D)** IGH and IGK gene rearrangements were detected. (M:Marker; P:Positive control; N:Negtive control; HC:IGHC; KA:IGKA).

**Figure 2:**
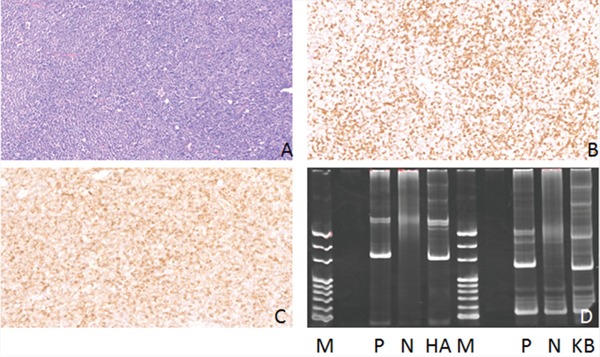
Hematoxylin-eosin(HE), Immunohistochemical staining and IG gene rearrangement of small lymphocytic lymphomas **(A)** Small lymphocytic lymphomas showed diffuse distribution of tumor cells (HE) (×200); **(B)** The lymphoma cells were positive for CD5 protein in the cytomembrane (×200); **(C)** The lymphoma cells were positive for CD23 protein in the cytomembrane (×200); **(D)** IGH and IGK gene rearrangements were detected. (M:Marker; P:Positive control; N:Negtive control; HA:IGHA; KB:IGKB).

**Figure 3:**
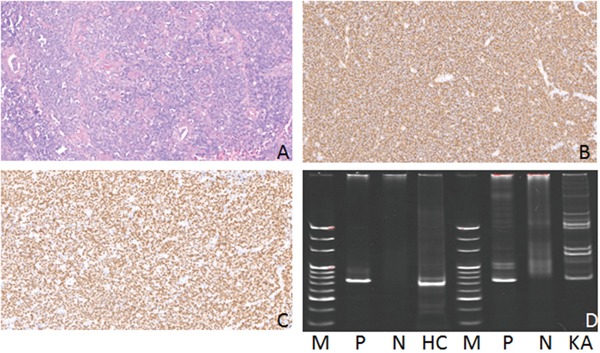
Hematoxylin-eosin(HE), Immunohistochemical staining and IG gene rearrangement of extranodal marginal zone B-cell lymphomas of mucosa-associated lymphoid tissue **(A)** Extranodal marginal zone B-cell lymphomas of mucosa-associated lymphoid tissue showed diffuse distribution of tumor cells(HE) (×200); **(B)** The lymphoma cells were positive for CD20 protein in the cytomembrane (×200); **(C)** The lymphoma cells were positive for PAX-5 protein in the nucleus (×200); **(D)** IGH and IGK gene rearrangements were detected. (M:Marker; P:Positive control; N:Negtive control; HC:IGHC; KA:IGKA).

**Figure 4:**
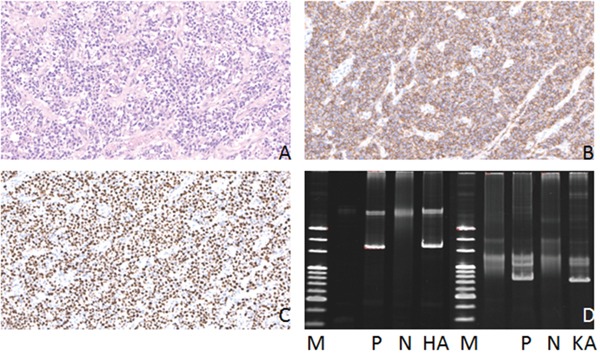
Hematoxylin-eosin(HE), Immunohistochemical staining and IG gene rearrangement of diffuse large B-cell lymphomas **(A)** Diffuse large B-cell lymphomas showed diffuse distribution of large tumor cells (HE)(×200); **(B)** The lymphoma cells were positive for CD20 protein in the cytomembrane (×200); **(C)** The lymphoma cells were positive for Ki-67 protein in the nucleus (×200); **(D)** IGH and IGK gene rearrangements were detected. (M:Marker; P:Positive control; N:Negtive control; HA:IGHA; KA:IGKA).

**Figure 5:**
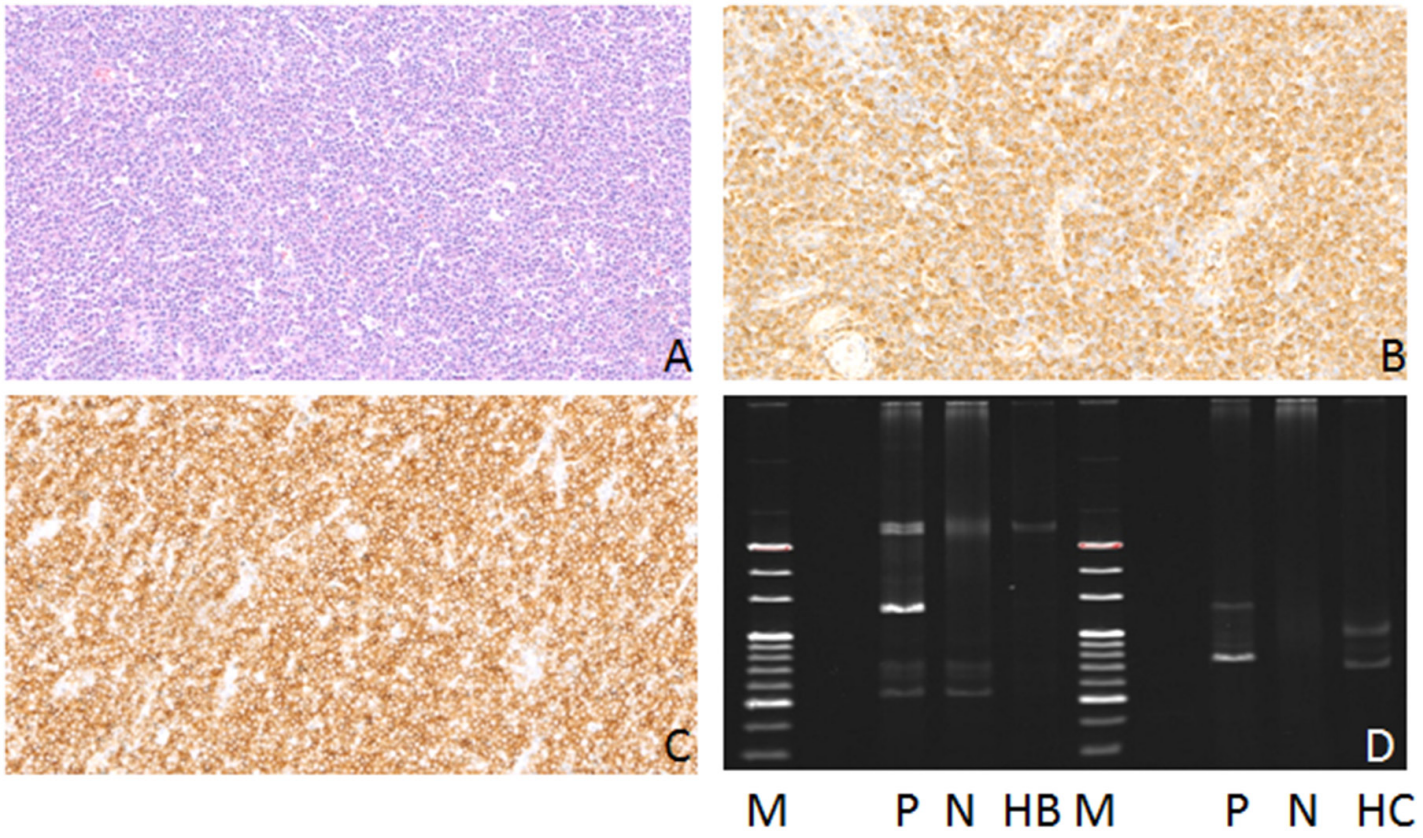
Hematoxylin-eosin(HE), Immunohistochemical staining and IG gene rearrangement of lymphoplasmacytoid lymphomas **(A)** Lymphoplasmacytoid lymphomas showed diffuse distribution of tumor cells(HE)(×200); **(B)** The lymphoma cells were positive for Kappa protein in the cytomembrane (×200); **(C)** The lymphoma cells were positive for CD38 protein in the cytomembrane (×200); **(D)** IGH gene rearrangements were detected. (M:Marker; P:Positive control; N:Negtive control; HB:IGHB; HC:IGHC).

**Figure 6:**
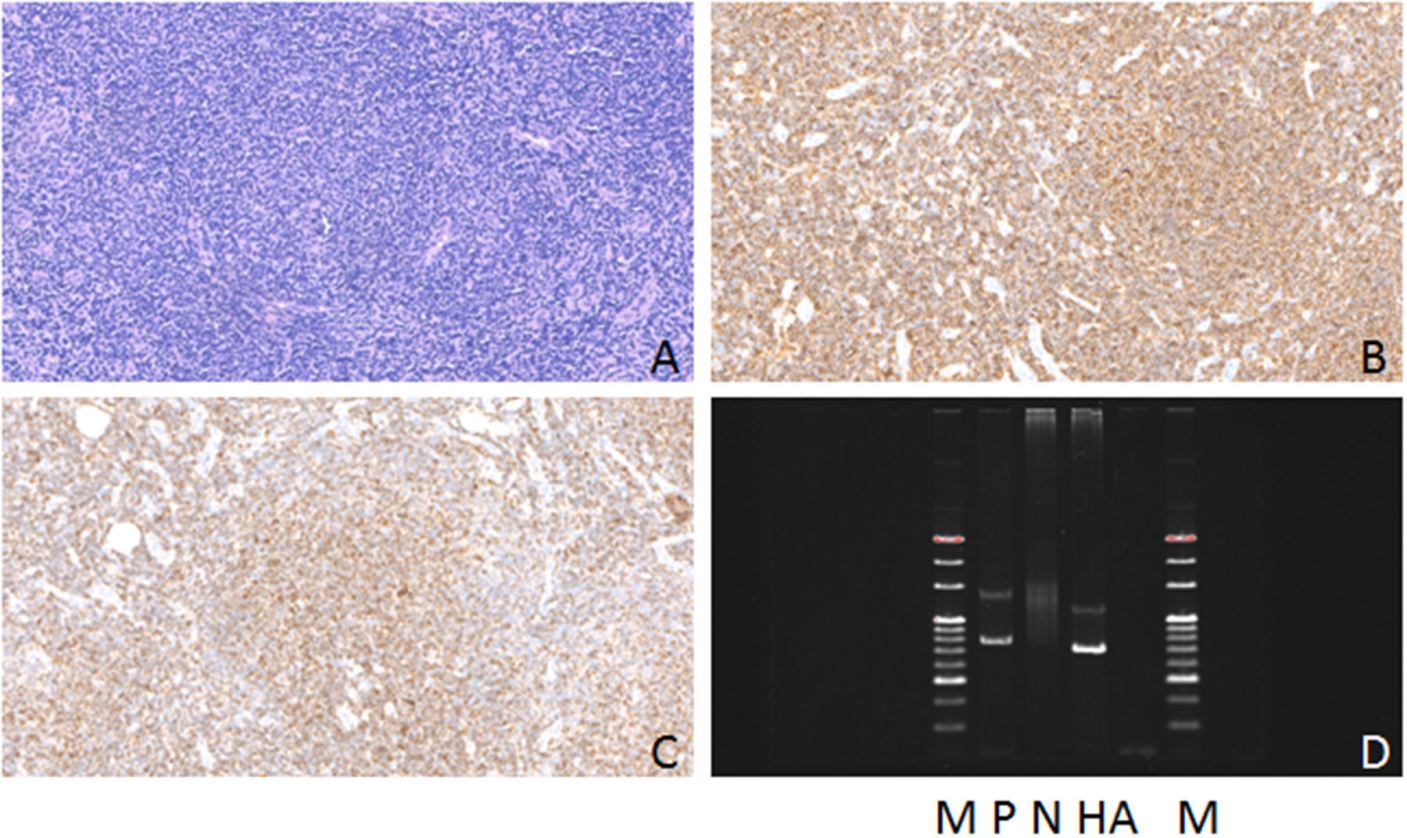
Hematoxylin-eosin(HE), Immunohistochemical staining and IG gene rearrangement of follicular lymphomas **(A)** Follicular lymphomas showed nodular distribution of tumor cells (HE)(×200); **(B)** The lymphoma cells were positive for CD20 protein in the cytomembrane (×200); **(C)** The lymphoma cells were positive for bcl-2 protein in the cytomembrane (×200); **(D)** IGH gene rearrangements were detected. (M:Marker; P:Positive control; N:Negtive control; HA:IGHA).

**Figure 7:**
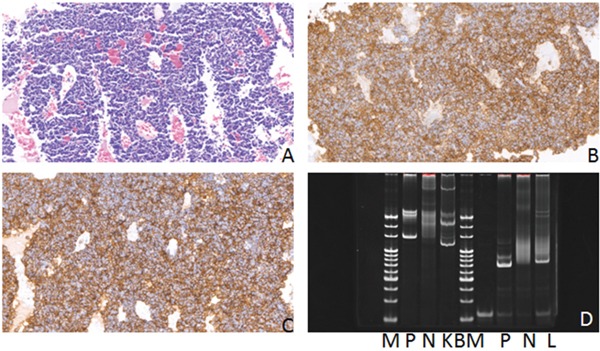
Hematoxylin-eosin(HE), Immunohistochemical staining and IG gene rearrangement of plasmocytomas **(A)** Plasmocytomas showed diffuse distribution of tumor cells (HE)(×200); **(B)** The lymphoma cells were positive for CD38 protein in the cytomembrane (×200); **(C)** The lymphoma cells were positive for CD138 protein in the cytomembrane (×200); **(D)** IGK and IGL gene rearrangements were detected. (M:Marker; P:Positive control; N:Negtive control; KB:IGKB; L:IGL).

**Figure 8:**
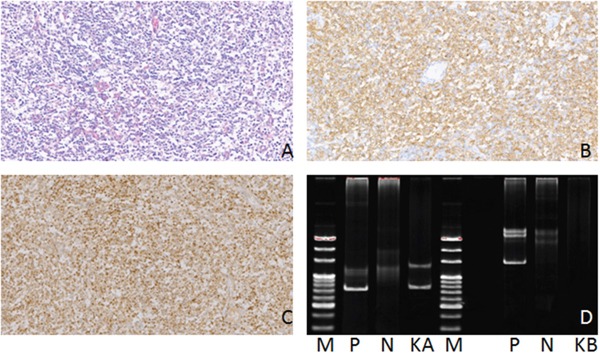
Hematoxylin-eosin(HE), Immunohistochemical staining and IG gene rearrangement of nodal marginal zone B-cell lymphomas **(A)** nodal marginal zone B-cell lymphomas showed diffuse distribution of tumor cells (HE)(×200); **(B)** The lymphoma cells were positive for CD20 protein in the cytomembrane (×200); **(C)** The lymphoma cells were positive for bcl-2 protein in the cytomembrane (×200); **(D)** IGK gene rearrangements were detected. (M:Marker; P:Positive control; N:Negtive control; KA:IGKA; KB:IGKB).

In the 209 cases of B-cell lymphoma analyzed, IGH and IGK gene rearrangements were mainly found in MCL (Figure 1), CLL (Figure 2), MALT (Figure 3) and DLBCL (Figure 4). IGH gene rearrangement was mainly found in LPL (Figure 5) and FL (Figure 6). IGK and IGL gene rearrangements were mainly found in plasmocytoma (Figure 7). IGK gene rearrangement was mainly found in MZL (Figure 8) (Table 2).

**Table 2:** Correlation between IG gene rearrangements and lymphoma classification

Lymphoma type	n	IGH	IGK	IGL	IGH+ IGK	IGH+ IGL	IGK + IGL	IGH+ IGK + IGL
MALT	69	56	53	31	43	26	23	20
DLBCL	63	51	51	32	41	26	28	23
FL	39	30	28	15	20	10	11	7
CLL	15	14	14	9	13	9	8	8
plasmocytoma	6	3	6	6	3	3	6	3
MCL	6	6	6	5	6	5	5	5
MZL	7	2	7	1	2	1	1	1
LPL	4	4	2	1	2	1	1	1
Total	209	167	167	100	129	81	83	68

## DISCUSSION

The B cell receptor (BCR) on the B cell membrane surface identifies and combines with antigens and mediates the immune response [8]. The BCR on the B cell membrane surface is immunoglobulin (IG) and is composed of heavy chains (H) and light chains (L). The regions of IG outside the cell membrane can be divided into the variable regions (V) and constant regions (C). The V region with its structural diversity is involved in the specific binding of antigens [9]. The diversity of the BCR is the outcome of IG gene rearrangements during the process of B cell development [10]. The H chain gene is composed of VH, DH, JH and CH genes, the L chain gene of K and λ genes, the K gene of VK, JK and CK genes, and the λ gene of Vλ, Jλ and Cλ genes. These genes are transcribed only after IG gene rearrangements are complete [11].

Gene rearrangement is a normal physiological process that occurs during B lymphocyte maturation. In the embryonic state, the B lymphocyte immunoglobulin gene is composed of a variable region (V), diverse region (D), joining region (J) and constant region (C). These regions are nonconsecutive on the chromosome and are separated by insertion sequences of different lengths. After a certain stage of lymphocyte development, these regions realign to assemble into a structural gene, i.e., they undergo gene rearrangements, in a process that is catalyzed by a recombinase. The rearranged gene of each lymphocyte is unique, and therefore, B-cells can recognize millions of different antigens. If a single cell can evade physiological regulation due to the presence of specific factors, it will grow uncontrollably in a process called cloning hyperplasia, and malignant lymphoma will ensue. This cloning hyperplasia can lead to special gene rearrangements becoming dominant, which can lead to the generation of a clone detection index for malignant lymphoma [12–21]. Malignant lymphoma is monoclonal hyperplasia, and theoretically, all clones represent IG gene rearrangements. However, hyperplasias of normal lymphoid and reactive lymphoid tissues involve polyclonal rearrangements of the IG gene. The detection of IG gene rearrangements can usually help determine the nature of the lesions from their monoclonal and polyclonal origin. Therefore, IG gene rearrangements can help distinguish between benign and malignant lymphocyte proliferation.

In 2003, 47 organizations from 7 countries in Europe developed the BIOMED-2 multiplex PCR system, which contains 107 primers divided into 18 multiplex PCR tubes [22]. This new system can increase the sensitivity and specificity of the detection of lymphocyte clone hyperplasia with gene rearrangements. Employing PCR to detect the IG receptor gene has a relatively low requirement in terms of quality and concentration of template DNA but shows relatively high sensitivity, and therefore, even 1% to 5% clonal rearrangements of tumor cells can be detected [23]. Therefore, this technique could be important for early diagnosis of lymphoma. With improvements in the primer system and experimental techniques, and especially the application of the BIOMED-2 primer system, the rate of early diagnosis of lymphoma has increased significantly.

In this study, the BIOMED-2 system was employed to analyze IG gene rearrangements in 244 samples of paraffin embedded lymphoid tissue. Of these, 209 B-cell lymphoma samples were positive for IG gene monoclonal rearrangement, but the 35 lymphadenosis samples did not show IG gene monoclonal rearrangement bands (false negatives and false positives were zero), suggesting that the detection rate was 100%, which is markedly higher than that of other detection systems [24, 25] and consistent with Evans's report [15].

To avoid false negatives, primers for the functional fragments of IGH, IGK and IGL gene were designed in the BIOMED-2 system. For B-cell lymphomas localized in the germinal center or precursor germinal center [26, 27], and especially for B-cell lymphomas with IGH antibody isotype switching (for example, follicular lymphoma, diffuse large B-cell lymphoma, etc.), false negatives for lymphoma IG gene amplification are often observed because of large numbers of mutations in body cells [28]. Not only a complete rearrangement (VH-JH) but also an incomplete rearrangement (DH-JH) was contained in the IGH gene locus. Incomplete IGH rearrangements (IGHB) were incorporated into the BIOMED-2 primer system to improve the detection rate. In this study, 94 IGHA clonal rearrangements were detected with a 44.8% detection rate. With the inclusion of IGHB target analysis, 135 IGH A/B clonal rearrangements were detected (detection rate 65%). With the inclusion of IGHC target analysis, 167 IGH A/B/C clonal rearrangements were detected (detection rate 80%). The improvement in the detection rate can be attributed to the fact that the large numbers of mutations in body cells had almost no effects on the rearrangement pattern of IGHB; therefore, the detection sensitivity of lymphomas is increased [29] but still lower than the detection levels in Abbas F's report [30].

With the introduction of the IGK light chain, 204 IGH/IGK clonal rearrangements were detected, indicating a detection rate of 98%. When V-J and Kde fragments were included in IGK, this rearrangement occurred in all IG λ positive and one third of the IG κ positive B-cell lymphomas [31–33]. Moreover, the detection rate of mature B-cell lymphomas improved further and was the same as that reported by Zhang J [33]. The detection rate of IGL clonal rearrangements is relatively lower; only 100 samples were detected (detection rate 48%), but this was still higher than that reported by Abbas F [30]. The difference between IGH, IGK and IGL rearrangement rates in B-NHL and that of benign reactive lymph node hyperplasia was significant, indicating that the analysis of IGH, IGK and IGL gene rearrangements had clinical value in confirming clonality and the origin of B-cell NHL, which could be utilized in the diagnosis and classification of lymphomas.

Furthermore, IGH and IGK gene rearrangements were mainly found in MCL, CLL, MALT and DLBCL. IGH gene rearrangement was mainly found in LPL and FL, IGK and IGL gene rearrangements primarily in plasmocytoma, and IGK gene rearrangement primarily in MZL. The differences in IGH, IGK and IGL gene rearrangement rates in DLBCL, MALT, FL, CLL, MCL, MZL, plasmocytoma and LPL indicated that analysis of the gene rearrangement rate could be clinically important in the classification of different B-cell NHLs.

The BIOMED-2 system required that the load of oncocytes be between 1% and 5%. If the number of oncocytes were too low, the monoclonal bands would not be clear in a polyclonal background [34]. The locations of the bands should be thoroughly examined while analyzing the results with the BIOMED-2 system. Only samples with special amplified bands that appear in the expected fragment range can be judged as positive. False positives can be distinguished by sing-strand conformation polymorphism, denaturing gradient gel electrophoresis, DGGE, TGGE, HPLC, heteroduplex analysis and sequencing methods. In this study, heteroduplex analysis was used to clearly distinguish monoclonal rearrangements (sharp-edged bands) and polyclonal rearrangements (daubed bands), which improved the sensitivity and specificity significantly.

In conclusion, a higher detection rate and specificity can be achieved by using the BIOMED-2 system to diagnose malignant B-cell tumors. However, a detailed understanding of the results would require new insights into lymphoma development. Additionally, when analysis of gene rearrangements is used to diagnose lymphoma, it is necessary essential that these data be combined with PCR results, clinical data, tissue morphology and immunohistochemical results to enable a comprehensive assessment of the disease.

## MATERIALS AND METHODS

### Case information

A total of 209 excised B-cell lymphoma samples collected by the pathology department at Xiangya Hospital from June 2014 to December 2016 were used for the study. Of the 209 cases, 126 were male and 83 female. The age of the patients from whom the samples were obtained ranged from 6 to 83 years (mean age 51). Following classification, there were 69 MALT lymphomas, 63 DLBCL, 39 FL, 15 SLL, 6 plasmacytomas, 6 MCL, 7 MZL, and 4 LPL. The control group was comprised of 35 cases of reactive lymphoid hyperplasia.

### Immunohistochemical experiments

All specimens were fixed in 10% neutral formalin, entrapped through conventional paraffin embedding, and processed into 4 μm serial sections with conventional HE staining. The immunohistochemical S-P method was employed to mark immune phenotypes. LCA (2B11, DAKO), CD3 (F7.2.38, DAKO), CD45RO (OPD4, DAKO), CD20 (L26, DAKO), CD10 (MX002, DAKO), CD15 (Carb-3, DAKO), CD30 (Ber-2, DAKO), Bcl-2 (SP66, DAKO), Bcl-6 (LN22, AKO), CyclinD-1 (DCS-6, DAKO), PAX-5 (SP34, DAKO), kappa light chain (L1C1, DAKO) and lambda light chain (LAM03+HP6054, DAKO) were used.

### Reagents

The IdentiClone™ B-Cell Clonality Assay kit was purchased from Invivoscribe Technologies. The 20 bp DNA ladder and 10× loading buffers were purchased from Takara. GelRed dye was purchased from BioTium, AmpliTaq Gold DNA polymerase from ABI, and DNA FFPE Tissue Kit from Xiamen Aide Biomedicine Limited.

### DNA extraction

Ten paraffin sections of 8 μm thickness were transferred to 1.5 mL sterile centrifuge tubes. Genomic DNA was extracted using the extraction kit, and an ultraviolet spectrophotometer was used to determine the concentration and purity of the DNA.

### Primer design

The BIOMED-2 primer system [35–37] was used for the analysis of IG gene clonal rearrangements. Following the BIOMED-2 guidelines, three sets of VH primers were designed with the help of the OLIGO 6.2 program corresponding to the three VH FR regions (FR1, FR2, and FR3). Each set of primers consisted of six or seven oligonucleotides capable of annealing to their corresponding VH segments (VH1–VH7) with no mismatches for most VH segments and one or at most two mismatches for some rare VH segments [38]. The following are the primer sets that were used. IGH tube A V_H_ family primers: GGCCTCAGTGAAGGTCTCCTGCAAG, GT CTGGTCCTACGCTGGTGAAACCC, CTTGGGGGGT CCCTGAGACTCTCCTG, CTTCGGAGACCCTGTCC CTCACCTG, CGGGGAGTCTCTGAAGATCTCCTGT, TCGCAGACCCTCTCACTCACCTGTG;

IGH tube B V_H_ family primers: CTGGGTGC GACAGGCCCCTGGACAA, TGGATCCGTCAGCCCC CAGGGAAGG, GGTCCGCCAGGTCCAGGGAA, TGG ATCCGCCAGCCCCCAGGGAAGG, GGGTGCGCCA GATGCCCGGGAAAGG, TGGATCAGGCAGTCCCC ATCGAGAG, TTGGGTGCGACAGGCCCCTGGACAA;

IGH tube C V_H_ family primers: TGGAGC TGAGCAGCCTGAGATCTGA, CAATGACCAACATG GACCCTGTGGA, TCTGCAAATGAACAGCCTGAG AGCC, GAGCTCTGTGACCGCCGCGGACACG, CAG CACCGCCTACCTGCAGTGGAGC, GTTCTCCCTGC AGCTGAACTCTGTG, CAGCACGGCATATCTGCAG ATCAG.

IGH tubes A, B AND C J_H_ primer: CCAGTG GCAGAGGAGTCCATTC.

IGK tubes A and B Vk family primers: TCAAG GTTCAGAGGCAGTGGATCTG, GGCCTCCATCTCC TGCAGGTCTAGTC, CCCAGGCTCCTCATCTATGAT GCATCC, CAACTGCAAGTCCAGCCAGAGTGCAT CC, CCTGCAAAGCCAGCCAAGACATTGAT, GACCGATTTCACCCTCACCCTCACAATTAATCC;

IGK tube A Jk primers: CCCTGGTTCCACCTC TAGTTTGCATTC, CCCTGTGCTGACCTCTAATTTG CATTC; IGK tube B Jk primer: CGTGGCACCGCGA GCTGTAGAC. IGL tube V_λ_ primers: V_λ_1/2 ATTCTCT GGCTCCAAGTCTGGC, V_λ_3 GGATCCCTGAGCGAT TXTXTGG; J_λ_ primer: CCCTGGTTCGAGTGGCAG GATC. There were also 5 pairs of BIOMED-2 internal control primers. To analyze the integrity of the extracted DNA, the amplified fragments with these primers should be 100, 200, 300, 400 and 600 bps long. The housekeeping gene β-actin was used as the internal control. The products of PCR were analyzed by polyacrylamide gel electrophoresis.

### Evaluation of results

Compared with the standard, samples that showed 1 or 2 unusual clear bands different from the background product in a polyacrylamide gel were considered as positive. Additionally, the following 4 judgment standards proposed by McCarthy were applied: (1) the width of the electrophoresis bands should not be more than 1 mm with straight sides; (2) the molecular weights of the electrophoresis products should be within the desired values; (3) if no smeared band and no primer dimer are found, the samples should not be regarded as negative but the result interpreted as caused by an abortion of PCR amplification; (4) all the other bands that exceed the molecular weight range are nonspecific bands [39–42].

## Abbreviations

HL: Hodgkin lymphoma
NHL: non-Hodgkin lymphoma
IG: immunoglobulin
MALT lymphoma: extranodal marginal zone B-cell lymphomas of mucosa-associated lymphoid tissue
DLBCL: diffuse large B-cell lymphoma
FL: follicular lymphoma
SLL: small lymphocytic lymphoma
MCL: mantle cell lymphoma
LPL: lymphoplasmacytoid lymphoma
MZL: nodal marginal zone B-cell lymphoma
